# A self-directed upper limb program during early post-stroke rehabilitation: A qualitative study of the perspective of nurses, therapists and stroke survivors

**DOI:** 10.1371/journal.pone.0263413

**Published:** 2022-02-04

**Authors:** Lay Fong Chin, Ingrid C. M. Rosbergen, Kathryn S. Hayward, Sandra G. Brauer

**Affiliations:** 1 Rehabilitation Centre, Tan Tock Seng Hospital, Singapore, Singapore; 2 Physiotherapy, School of Health and Rehabilitation Sciences, University of Queensland, Brisbane, Australia; 3 STARS Surgical Treatment and Rehabilitation Service, The University of Queensland, Brisbane, Australia; 4 STARS Surgical Treatment and Rehabilitation Service, Metro North Health, Brisbane, Australia; 5 Physiotherapy, Melbourne School of Health Sciences, University of Melbourne, Parkville, Australia; 6 Stroke Theme, Florey Institute of Neuroscience and Mental Health, University of Melbourne, Melbourne, Australia; 7 NHMRC Centre of Research Excellence in Stroke Rehabilitation and Brain Recovery, Florey Institute of Neuroscience and Mental Health, University of Melbourne, Melbourne, Australia; Monash University Malaysia, MALAYSIA

## Abstract

**Introduction:**

This study aimed to explore the perspective of nurses, therapists and stroke survivors on the performance of upper limb self-exercise and use outside therapy during early inpatient stroke rehabilitation.

**Methods:**

A descriptive qualitative approach was used in focus groups with nurses (n = 21) and therapists (n = 8), as well as in-depth semi-structured interviews with stroke survivors (n = 8) who were undergoing subacute inpatient stroke rehabilitation. Inductive thematic analysis of data was performed according to participant group.

**Results:**

Nurses and therapists perceived that stroke survivors played a central role in determining the success of a self-directed upper limb program. Nurses perceived that stroke survivors needed a lot of prompting to be motivated to perform self-directed upper limb therapy outside therapy. Therapists perceived that not all stroke survivors would be able to perform self-directed upper limb therapy and deemed it important to consider stroke survivor factors before commencing a program. Although some stroke survivors expressed initial reservations with performing self-practice, many indicated that they would participate in the self-directed upper limb program because they wanted to recover faster.

**Conclusion:**

A difference between the perspective of nurses/therapists and stroke survivors towards self-directed upper limb performance outside therapy was found. Deeper stroke survivor engagement and a shift in rehabilitation culture to encourage stroke survivor autonomy are important considerations for a self-directed upper limb program. Teamwork amongst healthcare professionals and families is essential to support stroke survivors to participate in a self-directed upper limb program during early inpatient stroke rehabilitation.

## Introduction

Meta-analyses of stroke rehabilitation trials that have targeted motor recovery show that increased therapy dose can improve functional outcome [1–3]. There appears to be a threshold of 240% extra therapy needed to reliably improve motor outcome [3]. Observational studies demonstrate that the amount of upper limb therapy during standard stroke rehabilitation is subthreshold [4–6].

To increase upper limb therapy dose, one option is to increase therapist resources. This approach is costly and likely to be unsustainable with rising demands on the healthcare system. An alternative option is for stroke survivors to complete self-directed programs which tap into stroke survivor and caregiver self-management. Such programs have been shown to increase the amount of upper limb exercise performed and improve functional outcomes [7, 8]. Besides upper limb self-exercises, promotion and guiding of early upper limb use in daily functional activities is important. However, this is particularly challenging for people with moderate and severe upper limb impairments as they do not have enough upper limb movement to work with and do not know what to do and how to exercise [9, 10]. Despite this challenge, the engagement of upper limb use in daily functional activities is important to prevent “learned nonuse” [11] further down the path of recovery after stroke. The use of the upper limb in functional activities has been minimally addressed in previous program such as the Graded Repetitive Arm Supplementary Program (GRASP) [7]. Hence, the **S**elf-Empowered **U**pper Limb **R**epetitive **E**ngagement (SURE) program comprises both self-exercise and promotion of upper limb use early stroke rehabilitation was developed by our team to increase upper limb practice dose to facilitate upper limb recovery during early rehabilitation.

An observational study has shown that paretic upper limb use outside therapy is low [12] and another study reported that stroke survivors in hospital spent <1% of their time performing self-directed exercises [13]. These findings showed that it is challenging to increase upper limb use outside therapy. When developing a new program, it is important to seek the opinions of main stakeholders (nurses, therapists and stroke survivors) who are key to support the implementation of self-directed upper limb therapy [14]. To our knowledge, no qualitative studies have examined the perspective of nurses, therapists and stroke survivors with regard to self-directed upper limb performance during early instroke survivor stroke rehabilitation. Therefore, the aim of this study was to explore the perspective of nurses, therapists and stroke survivors on the performance of self-directed upper limb therapy outside therapy during early post-stroke inpatient rehabilitation to understand the facilitators and barriers of SURE program implementation.

## Methods

### Study context and ethics

This study was a prelude to a randomized controlled pilot trial of SURE program (NCT03425890) [15]. The results of the current qualitative investigation were used to improve the SURE program in the pilot trial. This study was approved by the National Healthcare Group Domain Specific Review Board Ethics in Singapore (2016/00227) and University of Queensland, Australia (2017000834/2016/01036) and conforms with the Declaration of the Helsinki. All participants in this study provided written informed consent. The study was carried out in a subacute 90 bed rehabilitation centre in Singapore in which 50 beds are dedicated for stroke survivors. Usual clinical practice included both occupational therapy and physiotherapy sessions (approximately 45 minutes per session) per weekday. The guidelines of Consolidated Criteria for Reporting Qualitative Research [16] and the Standards for Reporting Qualitative Research [17] were followed in conducting and reporting this research study.

### Design

This study used a descriptive qualitative approach to understand the perspective of our participants. Focus groups were used to explore the perception of nurses and therapists on stroke survivors’ paretic upper limb use outside therapy during inpatient rehabilitation and the SURE program. Interactions within the focus groups encouraged the nurses and therapists (occupational therapists and physiotherapists) to interact with one another to clarify their individual and shared perspectives [16]. Individual in-depth semi-structured interviews with stroke survivors who were inpatients in the rehabilitation centre were conducted. The individual interviews allowed the exploration of personal experience of the stroke survivors and their perspective towards their paretic upper limb use outside therapy and SURE program [16].

### Participants

Nurses and therapists with different years of work experience and seniority who were involved in direct patient care working in the rehabilitation centre were purposively recruited to attend one of four focus groups (3 nurse groups and 1 therapist group including 6–8 participants in each group). Inpatients with a diagnosis of stroke due to infarct or haemorrhage (confirmed by MRI/Computed axial tomography scans) with moderate to severe upper limb impairment (Fugl Meyer upper limb score ≤50) [18] who were medically stable, able to follow single stage commands and receiving occupational and physiotherapy were invited to participate. Stroke survivors with severe aphasia, neglect, agitation, or delirium/dementia which resulted in an inability to give consent and/or may have resulted in poor participation in interviews were excluded. The planned number of nurse, therapist and stroke survivor participants to recruit was based on the intent to capture the perspective of approximately 20% of the total number of nurses (≈90), therapists (≈40) and stroke patients with moderate to severe upper limb impairment (≈30) engaged at the rehabilitation centre at one time to provide a representative sample.

### The investigators’ relationship with participants

The principal investigator (LFC) was a female physiotherapist who has been working with stroke survivors in the rehabilitation centre where the study was conducted for 18 years. Investigator LFC led the design of the SURE program together with other therapists and believed that increased paretic upper limb use may improve upper limb outcomes after stroke. LFC had a professional relationship with nursing staff and therapists and this study was part of her PhD. Due to a high turn-over of nursing staff in the rehabilitation centre, the majority of participating nurses (81%) were not familiar with LFC. However, LFC was a senior colleague to therapist participants. Therefore, another interviewer (CT) conducted the therapist focus group. CT was a female physiotherapist with 4 years of working with stroke survivors in the rehabilitation centre and was involved with LFC in designing the SURE program. Prior to the therapist focus group, LFC outlined the questions and the guidelines of conducting focus groups to CT. All stroke survivors who participated were not familiar with LFC as she was not involved in their rehabilitation care. The nurses’ focus groups and all individual stroke survivor interviews were conducted by LFC.

### Data collection

Focus groups and interviews were collected from March to April 2017. For focus groups, all potential participants were approached by LFC during their morning roll calls and people individually volunteered. Nurse and therapist focus groups were conducted in a quiet room in the ward and therapy gym respectively with only one investigator present during each focus group. The purpose of why the study was performed was explained to all participants at the beginning of each focus group.

For individual stroke participant interviews, stroke survivors who met the inclusion criteria were invited to participate in the study by LFC. Each interview was conducted in a quiet corner in the ward or therapy gym. Interviews were conducted in English or Mandarin depending on stroke participant understanding and ability to engage in conversation. The interview questions were translated and asked by LFC who was fluent in both languages. Interviews were conducted with the stroke participant alone or with a caregiver present. Key stimulus questions for the focus groups and interviews can be found in Table 1. Booklets of the proposed SURE program were shown to nurses, therapists and stroke participants. All participants were encouraged to give their opinions and responses spontaneously. The investigators encouraged participants to share their perspectives towards paretic upper limb use outside therapy and SURE program during the focus groups and interview. A summary of responses was provided to all participants at the end of each focus group and interview to ensure it reflected the content discussed. All focus groups and individual interviews were audio recorded. Notes were made by the investigator during the session to summarize the points made by the participants. Details in data collection were added to ensure transferability of results.

**Table 1:** Stimulus questions for nurse and therapist focus groups and stroke participant interviews.

Nurse/therapist focus groups	Stroke participant interviews
1) What does rehabilitation after stroke mean to you?	1) What does rehabilitation after stroke mean to you?
2) What do you think of patients with stroke using their stroke-paretic arm and hand to practice tasks and exercises on their own in the ward outside their therapy sessions?	2) What do you think of people with stroke like you using your weaker arm and hand to practice tasks and exercises on your own in the ward outside your therapy sessions?
3) We are proposing a new program called the SURE program. Explain SURE program. Booklets of SURE program will be shown to the focus group. The participants will be given 5–10 minutes to look through the booklets. What is your feedback on SURE program?	3) We are proposing a new program called the SURE program. Explain SURE program. Booklets of SURE program will be shown to the stroke participants. The participants will be given 5–10 minutes to look through the booklets. What is your feedback on SURE program?
4) What are the barriers/difficulties that you foresee that will make the SURE program unsuccessful or difficult to carry out in the ward?	4) Do you think you will participate in SURE program? If yes, why? If not, why not?
5) What are the improvements we can make to make SURE program successful and easier to be carried out in the ward?	5) What are the barriers/difficulties that you foresee that will make stop you from carrying out the SURE program?
6) In what ways do you think nurses/therapists can help with the implementation of the SURE program in the ward?	6)What are the improvements we can make to make SURE program successfu1 and easier for you to participate?

Demographic data of nurses and therapists collected included age, sex, occupation, seniority, number of years working as a clinician, and number of years working with stroke survivors. Demographic data of stroke participants were collected from medical records and included age, sex, diagnosis (i.e. infarct/haemorrhage), paretic side, days since stroke onset, days since admission to rehabilitation centre and upper limb impairment level (Fugl Meyer upper limb score). The Fugl Meyer upper limb assessment were performed by occupational therapists trained in performing this assessment via video.

### Data analysis

Audio recording of all focus groups and interviews were transcribed verbatim by professional transcription agency (Pacific Transcription, Australia). Mandarin interviews were translated and transcribed by Singaporean undergraduate students fluent at high school graduate level in Mandarin. The transcripts were not returned to participants for comments/correction. All transcripts were checked by LFC against the audio recordings to ensure accuracy. Inductive thematic analysis in which the themes were identified based on the data collected was used in this study [19]. Data analysis was performed separately for nurse, therapist and stroke participants. Iterative data analysis commenced from March 2017 and was completed by February 2019. To overcome potential bias, three additional team investigators (IR, SB, KH) who were not involved in conducting the focus groups and interviews, together with LFC were involved in the data analysis process. Firstly, the team investigators read through the transcripts independently to familiarize and get a sense of the data. Secondly, the team investigators went through the transcripts line by line independently to extract meaningful information and repeated patterns and topics. Thirdly, discussions were held among the team investigators to clarify data interpretation, collapse the data into categories and identify themes. Repeated discussions were conducted till consensus were reached among the team investigators on the themes and subthemes. The team then re-read the transcripts and extract quotations to ensure that the defined themes accurately reflect the participants’ perspectives. Further refinements of links and subthemes were made during the writing process to ensure consistency in defined themes. Credibility was enhanced with the repeated interactive discussions among the investigators to clarify data interpretation and themes identification. The elements of trustworthiness—credibility, transferability, dependability and confirmability [20] were used to enhance rigor in this study. The results of analysis were not communicated with the participants.

## Results

The targeted percentage of participant recruitment was met with over 20% of total nurses, therapists and stroke survivors with moderate and severe upper limb impairment included in this study. Twenty-one nurses (1 male and 20 females) and eight therapists (4 occupational therapists and 4 physiotherapists; 6 males and 2 females) participated in four focus groups (Table 2). The mean age of nurses was 36±13 years; working as a nurse for on average 10±9 years and working with stroke survivors for 9±9 years. The mean age of therapists was 32±5 years; working as a therapist for an average of 7±5 years and working with stroke survivors for 4±4 years. Ten stroke survivors were approached, two declined the invitation to participate and eight stroke survivors consented to be interviewed. Characteristics of stroke participants can be found in Table 3. The mean age of stroke participants was 59±13 years and the mean duration post-stroke was 31±17 days. Four interviews were conducted in Mandarin as the stroke participants did not understand English. The remaining four interviews were conducted in English. Six interviews were conducted with stroke participants alone and two stroke participants had caregivers present during the interviews. The duration of the focus groups ranged from 33 minutes to 61 minutes, and the individual interviews ranged from 8 to 18 minutes. No repeat interviews were conducted. For the nurses focus group, themes reached saturation point at the third focus group. For the therapist focus group, consistent themes arose but data saturation could not be confirmed with only one therapist focus group. For the stroke survivors, themes reached saturation with eight stroke survivor interviews.

**Table 2:** Characteristics of participants in focus groups.

	Age	Gender	Seniority	Clinical staff (years)	Working with stroke survivors (years)
*Nurses*					
N01	45	Female	SSN II	9	9
N02	24	Female	HCA	4	4
N03	29	Female	SN I	8	5
N04	27	Male	EN I	6	2
N05	32	Female	EN II	8	6
N06	62	Female	PEN	40	40
N07	28	Female	SSN II	8	8
N08	24	Female	SN II	1	1
N09	24	Female	EN I	4	4
N10	44	Female	SEN I	21	21
N11	33	Female	EN II	2	2
N12	31	Female	EN I	9	4
N13	28	Female	EN I	6	4
N14	52	Female	SSN II	12	8
N15	55	Female	SHCA	9	9
N16	32	Female	SHCA	10	10
N17	58	Female	EN I	20	18
N18	23	Female	EN I	5	5
N19	57	Female	SEN II	16	16
N20	28	Female	EN I	7	3
N21	23	Female	SN II	2	2
*Therapists*					
OT1	26	Female	OT II	3	3
OT2	37	Male	Senior OT I	12	12
OT3	31	Male	Senior OT II	6	4
OT4	40	Male	Principal OT II	16	7
PT1	27	Female	Senior PT II	5	2
PT2	27	Male	PT II	3	3
PT3	31	Male	Senior PT II	7	5
PT4	33	Male	PT II	1	1

Abbreviations: OT: Occupational Therapist, PT: Physiotherapist

Seniority of nurses (higher to lower position)

Senior Staff Nurse I (SSN I), Senior Staff Nurse II (SSN II), Staff Nurse I (SN I), Staff Nurse II (SN II), Principal Enrolled Nurse (PEN), Senior Enrolled Nurse (SEN), Enrolled Nurse I (EN I), Enrolled Nurse II (EN II), Senior Health Care Assistant (SHCA), Health Care Assistant (HCA)

Seniority of therapists (higher to lower position)

Principal Therapist I (Principal OT/PT I), Principal Therapist II (Principal OT/PT II), Senior Therapist I (Senior OT/PT I), Senior Therapist II (Senior OT/PT II), Therapist I (OT/PT I), Therapist II (OT/PT II).

**Table 3:** Characteristics of stroke participants.

	Age	Male/ Female	Type of stroke	Paretic side	Days since stroke	Days since rehabilitation admission	Fugl Meyer Upper Limb score
P01	36	Female	Infarct	Right	13	5	19
P02	53	Female	Haemorrhage	Left	56	20	16
P03	52	Male	Infarct	Left	18	8	4
P04	56	Male	Infarct	Right	53	33	11
P05	61	Male	Infarct	Left	31	14	6
P06	69	Female	Infarct	Left	37	32	7
P07	63	Male	Infarct	Right	14	5	50
P08	80	Female	Infarct	Left	28	21	34

After four discussion meetings for nurse focus group, three for therapist focus group and two for stroke participants’ interviews, consensus on final themes was obtained and outcomes of data analysis was documented as results of this study. The summary of themes and subthemes are presented in Table 4.

**Table 4:** Summary of the themes and subthemes from the nurse and therapist focus groups and from stroke participants interviews.

Nurses focus groups	Therapist focus group	Stroke survivor interviews
**1) The stroke survivor plays an important role in the success of the program**	**1) Guiding the stroke survivor on the journey**	**1) Stroke survivors’ attitude towards self-directed** upper limb **therapy is positive**
Stroke survivor motivation is critical	Recovery begins with rehabilitation	Stroke survivors want to do self-directed therapy because they want to get better
Many factors will negatively impact on adherence	Self-directed therapy to increase intensity and influence neuroplasticity	Time in the ward is “wasted time”
Nurses perception of stroke survivors’ attitude towards self-directed therapy outside therapy		Stroke survivors have reservations but are willing to participate in SURE program
Nurses can be a facilitator or a barrier		
**2) How to make the program work well**	**2) The stroke survivor needs to take charge**	
Delivery of intervention	Empower stroke survivors to take charge of their rehabilitation journey	
No added chores	Create a culture to enable stroke survivor ownership	
Everyone in the team has a role to play		
Bring the family members in		
Finding the correct timing		
** **	**3) Putting it into practice**	
	Stroke survivor factors	
** **	Delivery of intervention	
	Design of the program	

### Nurses’ perspectives

#### Theme 1. The stroke survivor plays an important role in the success of the program

Across all focus groups, nurses consistently indicated that stroke survivors play an integral role to the success of performing self-directed upper limb therapy outside therapy.

*Stroke survivor motivation is critical*. Nursing staff reported that success in performing self-directed upper limb therapy was dependent on the individual stroke survivor’s motivation, willingness and adherence. Nurses were skeptical that stroke survivors would initiate and practice exercises on their own and expressed stroke survivors would likely need to be encouraged and prompted.

“*Need to be prompt and motivated*, *they need a lot of motivation*. *They will not do it on their own*.*” N19*

*Many factors will negatively impact on adherence*. Nurses were relatively negative about the ability of stroke survivors to complete self-directed upper limb therapy, identifying numerous factors likely to impede adherence. Nurses perceived that physical factors such as tiredness, medical issues and pain would limit adherence to performance of self-directed upper limb therapy. Nurses also expressed that stroke survivors looked too tired after therapy to perform self-directed upper limb therapy and were concerned stroke survivors would have reduced rest time before the next therapy session.

Psychological factors such as low mood, lack of self-confidence and reduced attention were also suggested to negatively impact on the ability of stroke survivors to perform self-directed upper limb therapy. Nurses perceived that stroke survivors required education, guidance and frequent encouragement to enable engagement in self-directed therapy.

“*When they come up to the ward*, *they look so tired*, *exhausted and they want just to sleep*.*” N03*

*Nurses perception of stroke survivors’ attitude towards self-directed therapy outside therapy*. Nurses expressed that in the mind of stroke survivors there appeared a clear demarcation of what they were expected to do in the ward versus during therapy time. Nurses perceived that stroke survivors expected assistance from nurses in the ward, while during therapy they would work hard under the therapists’ directions. Nurses perceived that stroke survivors might find performing self-directed therapy in the ward to negatively impact their therapy time as it might leave them too tired.

“*I feel these patients when they finish their therapy they come up here*, *their mindset it goes like*, *now it’s your duty*, *you’ll do for me*, *I am going to just relax*…*” N17*

*Nurses can be a facilitator or a barrier*. Nurses identified that they could play an important proactive role to support stroke survivors performing self-directed upper limb therapy. Nurses felt that they could facilitate stroke survivors to complete upper limb therapy by showing the stroke survivors the purpose, giving them direction and building hope that if they work harder it would enhance recovery. Nurses expressed that they could facilitate by reminding, following up, encouraging, supporting stroke survivors to use their paretic upper limb and perform self-exercises. On the other hand, many nurses also expressed concerns that stroke survivors would not be motivated to perform the exercises on their own and this could be a barrier.

“*…give them a kind of hope that if you work harder on the affected side you will improve*. *You must give them some direction and hope and how to do it” N01*

#### Theme 2. How to make the program work well

To make the program work well, a few factors need to be considered in program delivery with no added chores to the nurses/therapists, everyone working as a team, bringing the family in and finding the correct timing for the stroke survivors to perform the self-exercises.

*Delivery of intervention*. Nurses expressed that stroke survivors need to understand the purpose of self-directed therapy in order to be motivated. The self-exercises had to be simple and produce results, so stroke survivors and their caregivers were able to learn and perform them and see improvement in function. To support a successfu1 program, goal setting and a reward system to encourage stroke survivors to perform exercises were suggested.

“*…the exercises have to be simple ones for the patient*. *So if it’s simple*, *then they learn it and then their carers would actually learn it*.*” N08*

*No added chores*. For self-directed upper limb program to be successfu1, the nurses indicated that it must be incorporated into routine and cannot be viewed as an extra chore. Stroke survivors and/or families need to take ownership of the program, with the environment set up to make practice the easy choice (i.e. no extra task).

“*For it to be successful the staff cannot see it as extra chores so the planning in the first place should not be for staff to monitor*. *It should be the patient or family*…*” N01*

*Everyone in the team has a role to play*. Nurses expressed the importance to involve the whole rehabilitation team to complement and support each other. Therapists and nurses would need to ‘sing the same song’ in front of the stroke survivors, so stroke survivors understand that therapists and nurses collaborate to support stroke survivors in performing self-directed therapy outside therapy. Nurses perceived that therapists should be the first healthcare professional to initiate and educate the stroke survivors in the exercises, and to provide regular feedback to the stroke survivors to let them know if they were performing the exercises correctly. Nurses expressed that they could help supervise stroke survivors doing self-exercises during weekends when there was no therapy. In order for the nurses to be competent in teaching and correcting the exercises, they felt they needed to be trained.

“*Most important is the nurses and the therapists must really like…sing the same song…in front of the patient*.*” N17*, *N19*, *N21*

*Bring the family members in*. Nurses indicated it was critical to educate the family to be aware and support the stroke survivor in performing self-directed upper limb therapy. Family could remind, reinforce, assist and encourage stroke survivors to do self-exercises and use their paretic upper limb in functional activities in the ward.

“*Because sometimes the carer is the one sometimes reminding them because not all the time the nurse is there” N05*

*Finding the correct timing*. Nurses reported that there was a need to find the correct time for stroke survivors to perform self-directed therapy in the ward. Nurses strongly agreed that weekends would be the best time for self-directed upper limb therapy as stroke survivors could perform the self-exercises and use the paretic upper limb with the assistance of visiting family members.

“*I think during weekends there’s a possibility…they will do these exercises because no therapy*, *then no activities and pressure they will be doing it and then their family members to encourage and motivate them*.*” N03*

### Therapists’ perspectives

#### Theme 1. Guiding the stroke survivor on the journey

Rehabilitation was perceived as the beginning of rehabilitation journey with the therapists guiding the stroke survivor. Self-directed therapy was expressed by therapists as a means to increase practice intensity and influence neuroplasticity for recovery.

*Recovery begins with rehabilitation*. Therapists perceived that life after stroke was a journey that began with rehabilitation. Rehabilitation was viewed as not just the period of time in hospital but continued for many years after stroke as stroke survivors adapted to new challenges- physically and psychologically. Therapists perceived their role was to help guide the stroke survivors and their family along this journey, which included provision of support, education and knowledge sharing. In their opinion, a good rehabilitation centre need to find balance between maximizing recovery potential after stroke and adaptation strategies, compensation, equipment and device need for each individual stroke survivor. Rehabilitation involved everyone in the team including doctors, nurses, therapists, psychologists and social workers.

“*To give them the support that they need*, *the education*, *the knowledge that we can share with them as well as how they can best adapt to life after stroke as well*.*” PT1*

*Self-directed therapy to increase intensity and influence neuroplasticity*. Therapists supported the performance of self-directed upper limb therapy outside therapy as part of the rehabilitation journey because it could increase intensity, which in turn was perceived positive to possibly influence neuroplasticity and motor recovery after stroke. Therapists reflected that 45 minutes of daily therapy did not allow stroke survivors to practice sufficiently to influence neuroplasticity and motor recovery. If nurses and family supported self-directed upper limb therapy outside therapy and over the weekend, then perhaps higher intensity levels could be reached.

“*…we are seeing them maybe 45 minutes*. *So it cannot hit the repetition that we want the patient to do*. *So by doing all that exercise*, *using an ADL (activities of daily living)*, *family or nurses is helping*. *Asking the patients to using their weaker side in ADL*. *Then perhaps they will hit the timings to enhance the recovery*.*” OT3*

#### Theme 2. The stroke survivor needs to take charge

Therapists perceived that stroke survivors need to take charge of their own rehabilitation journey.

*Empower stroke survivors to take charge of their rehabilitation journey*. Therapists perceived that the stroke survivor was the most important member of the rehabilitation team who needed to be empowered to take charge of their own rehabilitation. This involved setting of meaningfu1 goals so that stroke survivors could self-drive practice. Therapists reflected that embedding a self-directed upper limb program at the beginning of the recovery journey was a good initiative to inculcate in the stroke survivors the value of taking responsibility of their paretic upper limb.

“*…the most important thing is for self-empowerment of the patient itself*. *But sometimes if they do not take charge of their rehabilitation process*, *outcomes will not be as good*.*” OT2*

*Create a culture that enables stroke survivor ownership*. Therapists perceived that nurses could facilitate this culture by prompting and encouraging stroke survivors to use their paretic hand to perform daily tasks instead of assisting stroke survivors too quickly. Therapists reflected that when behavioral change could be achieved while stroke survivors were inpatients, it might potentially lead to a better self-management in exercise routines after stroke survivors were discharged.

“*That we hope the patients can take charge of their own lives and have some sense of empowerment in terms of the future and in terms of what they should do*… *If they are given the autonomy to take up their own upper limb exercises*, *I think it’s a good initiative to inculcate at the very start of their journey*.*” OT1*

#### Theme 3. Putting it into practice

Therapists felt that it was important to consider stroke survivor factors before enrolment, and that the SURE program needed to be meaningful for the stroke survivors.

*Stroke survivor factors*. Therapists reported that it was important to consider stroke survivor factors before providing a self-directed upper limb program, as it might not be suitable for all stroke survivors. Therapists perceived that stroke survivors might be too tired after their daily therapy/multiple appointments and might not have the endurance and/or time to perform self-directed therapy. Therapists also perceived that stroke survivors with cognitive deficits may not be able to remember all the exercises. Therapists expressed that there were also stroke survivors who just simply did not want to perform exercises in the ward.

“*…I do believe that the patients that we chose to enroll into this self-empowered upper limb repetitive engagement program likely to be those—I mean assume*, *those who are already quite self-driven*, *cognitively*. *Maybe no severe cognitive deficits or visual perceptual deficits*.*” OT4*

*Delivery of intervention*. To comply with self-directed upper limb therapy, therapists emphasized that exercises or activities had to be meaningful and relevant to stroke survivors to enhance adherence. Therapists perceived that they needed to instruct the exercises to stroke survivors first, followed by regular follow-up. This was perceived to be particularly important during the initial phase of the program to prevent compensation. Therapists perceived that nurses could encourage, create awareness and remind stroke survivors to use their paretic upper limb in the ward. For nurses and families to be involved, therapists perceived that both nurses and family would need to be trained.

“*So as long as there’s something that is—the exercise or the activity is more meaningful*, *patients will do it more willingly*, *and it’s easier for them to remember*.*” OT2*

*Design of the program*. Therapists suggested not to restrict self-directed therapy to ‘just the program’ and to allow stroke survivors to add upper limb activities they found useful and were interested in. This results in self-directed therapy being more individualized. Therapists also suggested that the SURE booklet could be used as a communication tool between different professionals. Technology such as a game app on the stroke survivors’ mobile phone or gamification was suggested to motivate the stroke survivors and track their performance.

“*…may be good to have a selection of other upper limb activities for patient to choose*. *Or the patient may want to add on for some personal stuff aside*, *apart from the list*. *So it may be good to have an empty space for them allow to for addition*, *for changes*, *so it’s more personalized*. *It’s more individualized*.*” OT4*

### Stroke survivors’ perspectives

#### Theme 1. Stroke survivors’ attitude towards self-directed upper limb therapy is positive

Overall, stroke survivors were positive towards getting involved in SURE program.

*Stroke survivors want to do self-directed therapy because they want to get better*. Stroke survivors expressed that the program could facilitate them to learn to exercise and use their paretic upper limb outside therapy so that they may recover faster.

“*It’s good*, *I think it’s good… You can exercise and you can learn something from it*. *In addition*, *you can learn how to move your hand*.*” P04*

“*I mean*, *this is my own arm*, *I want to help it to get better faster*.*” P02*

*Time in the ward is “wasted time”*. Stroke survivors expressed that they had a lot of free time whilst in the ward and perceived this time as “wasted”. Stroke survivors felt they could use this time (especially weekends) to perform self-directed therapy, rather than watching television or sleeping.

“*I think it’s helpful because I have so much free time*, *especially on the weekends*, *and I have so many family members to help me out*, *why not*?*” P02*

“*If I want to get better*, *I have to practice more*. *Otherwise*, *I will be sleeping in the ward the entire day and that would not be of any use*.*” P06*

*Stroke survivors have reservations but are willing to participate in SURE program*. Although stroke survivors reported to see the benefits of a self-directed program, they highlighted individual reservations towards performing self-directed therapy on their own. Some stroke survivors even expressed fear in doing self-exercises outside therapy because they thought it could increase pain in their paretic upper limb. In general, stroke survivors perceive that they were not confident that they could perform the self-directed therapy correctly and felt they needed guidance and feedback from therapists to ensure correct performance. Although, stroke survivors expressed initial reservations towards performing self-directed therapy outside therapy, they indicated that they would participate in SURE program because they wanted to recover faster.

“*I wouldn’t dare to do it*, *I am afraid of moving my right hand… Pain*, *I am afraid of pain*. *Sometimes when it is painful inside*, *it will be painful*.*” P04*

## Discussion

This study sought to explore the perspective of nurses, therapists and stroke survivors regarding self-directed upper limb therapy performance outside therapy during early inpatient rehabilitation and to understand the facilitators and barriers to implementing a self-directed upper limb program in Singapore. Both nurses and therapists agreed that stroke survivors played a central role in determining the success of the self-directed therapy program. Overall, nurses perceived that stroke survivors would require a lot of prompting and encouragement to perform self-directed upper limb therapy and foresaw barriers. Therapists perceived that not all stroke survivors would be able to perform self-directed therapy and deemed it was important to consider stroke survivor factors before enrolling them into a self-directed program. Both nurses and therapists perceived that stroke survivors would be too tired to want to perform self-directed upper limb therapy outside therapy. However from the stroke survivors’ perspective, although some expressed initial reservations, all indicated that they would participate in self-directed upper limb exercises because they wanted to expedite their recovery. It is worthy to note that the themes from the stroke survivors were relatively consistent, reflecting a strong and persistent perspective among the stroke survivors towards self-directed upper limb therapy performance. From the results it can be seen that there is a difference in perspective between the nurses/therapists and the stroke survivors towards the performance of self-directed therapy outside therapy. Such difference in perspective between healthcare professionals and stroke survivors during rehabilitation is not new. In a previous study, healthcare professionals perceived that stroke survivors’ internal motivation was key, while stroke survivors identified external feedback and encouragement from healthcare professionals as a key motivator to drive their recovery after stroke [21]. This difference in perspective signifies a potential gap in understanding between the healthcare professionals and stroke survivors, which may impact the efficiency and effectiveness of rehabilitation. To fill this gap, there is a greater need for deeper consideration and greater stroke survivor engagement in rehabilitation program development [22–24].

Greater stroke survivor engagement is important because stroke survivors move suddenly to a state of dependency and lose control of their daily lives when they suffer a stroke [22] and become largely dependent on healthcare professionals to help and guide them in their rehabilitation journey. Thus, it is important to create a culture of empowerment and autonomy for the stroke survivors during rehabilitation as indicated by the therapists. Empowerment and autonomy in stroke survivors can be achieved by building trusting relationships with healthcare professionals [22] and engaging stroke survivors early in rehabilitation. It has been found in a previous study that when the stroke survivors and caregivers were actively involved in a rehabilitation program, it led to more tailored exercises and individualization of rehabilitation [25]. Tailored exercises and skills were then found to help stroke survivors and caregivers to be prepared for the home situation after discharge [25]. Taking stroke survivors’ engagement a step further, clinician can include stroke survivors in the coproduction of new rehabilitation programs [14]. Engaging stroke survivors in coproduction increases the stroke survivors’ confidence, empowers them to take control of their health and ultimately leads to “healthier behaviors” and improved outcomes [26]. Both stroke survivors and healthcare professionals need to be equipped with new skills and knowledge to enable coproduction to happen [27].

Stroke survivors perceived a lot of time in the ward was wasted and wanted to use this time to do self-exercises to hasten their recovery. This perception of “wasted time” is similar to a previous study in which stroke survivors described time outside therapy as “dead and wasted”, as well as bored and underexploited for recovery [21]. This finding concurs with an observational study which reported that stroke survivors are alone and inactive outside therapy [28]. This supports the notion that time outside therapy should be capitalized for self-directed therapy to optimize upper limb recovery after stroke. Weekends were raised among the nurses as an opportune time for self-directed therapy performance as there were fewer therapeutic activities and visiting family members could encourage and assist in self-directed upper limb therapy performance. The nurses even expressed that they could supervise stroke survivor self-exercise performance during weekends. This idea is supported by a previous study which showed that a nurse-led weekend group exercise program was both feasible and sustainable [29].

Notably, the results of the study revealed that for the program to be sustainable, it is crucial for it to be part of a stroke survivors daily routine and cannot add chores for nurses and therapists. While introducing upper limb self-exercise and daily upper limb use into stroke survivors’ daily routine promotes good habits formation and empowers stroke survivors to take ownership of their daily lives [21], this feedback also signifies the constraints in therapist resources in introducing any new innovative program in a real clinical world. Inadequate staffing and competing priorities in current post-stroke rehabilitation have been identified as barriers to the implementation of a large international A very early rehabilitation trial (AVERT) trial [30]. Perhaps there is a need to examine and prioritize rehabilitation processes to re-organize therapist workload to implement value based care to improve stroke survivor outcomes. Healthcare professionals need to identify ways to work manage barriers of implementation. With identified challenges, it appears crucial that the whole team of stroke survivors, families and healthcare professionals need to come “on board” and work together. Teamwork of close collaboration is seen as “central to success” in any new program implementation in stroke rehabilitation [30, 31]. Nurses and therapists can work closely (i.e. “sing the same song”) to nurture stroke survivors’ motivation through encouragement, support and feedback [22]. For teamwork to work, team members need to be “on board” which requires careful planning, education and monitoring [30].

Family is a precious resource and forms part of the team to support, assist and encourage stroke survivors in self-directed upper limb therapy outside therapy. Family has been found willing to help stroke survivors with their exercises in a previous study [32]. Family involvement has been found to enhance individualization of a treatment plan [25], increase time spent in upper limb self-exercise performance [8], improve upper limb function [8] and make family and stroke survivors more aware and ready in coping with home situation after discharge [25]. However, it is acknowledged that ongoing high-quality research is required. Together with the family, the whole team can collaborate to empower stroke survivors to take ownership of their participation in SURE program, their daily lives and recovery.

### Improving the SURE program

The main result of our study signifies a gap in understanding between healthcare professionals and stroke survivors towards self-directed upper limb therapy performance, thus showing that there is a need for greater engagement of stroke survivors in carrying out self-directed exercises and use via the SURE program. As a result of this study, the SURE program pilot trial actively involved stroke survivors in the program. This included actively listening to stroke survivors’ needs and problems in choosing the appropriate exercises/functional activities, as well as understanding what activities worked (or not) during reviews.

As a result of this study, the tested SURE program focused on making upper limb exercises/functional activities simple, meaningful and relevant so that the stroke survivors could manage the additional therapy dose and continue to be motivated to perform SURE program. In term of delivery, one day was added to performing self-directed therapy (from five to six days per week) to include at least one weekend day to capitalize on lower therapeutic activity on the weekend and potential involvement of visiting family members. For the SURE program to be sustainable, the program was integrated into the stroke survivors’ normal daily routine without adding to the workload of nurses and therapists.

### Limitations

Focus groups and interviews were conducted prior to the pilot trial of the self-directed upper limb program, hence perspectives of nurses, therapists and stroke survivors were based on their past experiences or their “postulations” of what would happen with the program implementation. It will be of value and interest to learn of the nurses, therapists and stroke survivors’ perspectives after they worked with the self-directed upper limb program.

Nevertheless, the results of this study provided valuable information on how to improve the self-directed upper limb program. While we confirmed saturation in the nurse and stroke survivor group, this was not confirmed in the therapist group, however we did identify consistent themes. Another limitation was that the majority of stroke survivors who expressed their views in this study had shown interest in participating in the self-directed upper limb program and thus represent a biased population. Two stroke survivors who rejected the invitation to participate were not interested in self-directed upper limb performance outside therapy. Hence, the perspective of stroke survivors not interested in self-directed upper limb performance might not be equally represented in this study. Further studies should seek perspective of those uninterested stroke survivors in order to gain a more comprehensive understanding on the stroke survivor perspective.

## Conclusions

Results of this study show that there was a difference between the perspective of healthcare professionals and stroke survivors towards self-directed upper limb performance outside therapy during early post-stroke rehabilitation. Such differences show that implementation of a self-directed upper limb program involves more than just “dishing out” self-exercises to stroke survivors outside therapy. It involves deeper stroke survivor engagement to empower the stroke survivors to have greater ownership in their recovery after stroke. This will likely require a shift in current rehabilitation culture to bring about an equal partnership, on-going communication and greater team work amongst healthcare professionals, stroke survivors and their families.

## Supporting information

S1 File Consolidated criteria for reporting qualitative research checklist. (DOCX)

S2 File Standards for reporting qualitative research checklist. (DOCX)
