# Supplementary material for: A self-directed upper limb program during early post-stroke rehabilitation: A qualitative study of the perspective of nurses, therapists and stroke survivors
Source: PLoS One. 2022 Feb 4;17(2):e0263413. doi: 10.1371/journal.pone.0263413 (PMC8815971; doi:10.1371/journal.pone.0263413)
Supplement: S1 File — (DOCX) [file pone.0263413.s001.docx]

| Consolidated criteria for reporting qualitative studies (COREQ): 32-item checklist No Item Guide questions/description | | |
| --- | --- | --- |
| 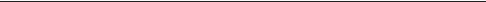   \| **Domain 1: Research team and reflexivity** \| \| --- \| |  |  |
| Personal Characteristics |  | page number |
| 1. Interviewer/facilitator | Which author/s conducted the interview or focus group? | 7 |
| 2. Credentials | What were the researcher’s credentials? E.g. PhD, MD | 7 |
| 3. Occupation | What was their occupation at the time of the study? | 7 |
| 4. Gender | Was the researcher male or female? | 7 |
| 5. Experience and training | What experience or training did the researcher have? | 7 |
| Relationship with participants |  |  |
| 6. Relationship established | Was a relationship established prior to study commencement? | 7 |
| 7. Participant knowledge of the interviewer | What did the participants know about the researcher? e.g. personal goals, reasons for doing the research | 7, 8 |
| 8. Interviewer characteristics | What characteristics were reported about the interviewer/facilitator? e.g. Bias, assumptions, reasons and interests in the research topic | 7 |
|  |  |  |
| **Domain 2: study design** |  |  |
| Theoretical framework |  |  |
| 9. Methodological orientation and Theory | What methodological orientation was stated to underpin the study? e.g. grounded theory discourse analysis, ethnography, phenomenology, content analysis | 6 |
| Participant selection |  |  |
| 10. Sampling | How were participants selected? e.g. purposive, convenience, consecutive, snowball | 6 |
| 11. Method of approach | How were participants approached? e.g. face-to-face, telephone, mail, email | 8 |
| 12. Sample size | How many participants were in the study? | 11 |
| 13. Non-participation | How many people refused to participate or dropped out? Reasons? | 11 |
| Setting |  |  |
| 14. Setting of data collection | Where was the data collected? e.g. home, clinic, workplace | 7, 8 |
| 15. Presence of non-participants | Was anyone else present besides the participants and researchers? | 7, 8 |
| 16. Description of sample | What are the important characteristics of the sample? e.g. demographic data, date | 13, 14 |
| Data collection |  |  |
| 17. Interview guide | Were questions, prompts, guides provided by the authors? Was it pilot tested? | 8, 9 |
| 18. Repeat interviews | Were repeat interviews carried out? If yes, how many? | 12 |
| 19. Audio/visual recording | Did the research use audio or visual recording to collect the data? | 10 |
| 20. Field notes | Were field notes made during and/or after the interview or focus group? | 8 |
| 21. Duration | What was the duration of the interviews or focus group? | 12 |
| 22. Data saturation | Was data saturation discussed? | 12 |
| 23. Transcripts returned | Were transcripts returned to participants for comment and/or correction? | 10 |
|  |  |  |
| **Domain 3: analysis and findings** |  |  |
| Data analysis |  |  |
| 24. Number of data coders | How many data coders coded the data? | 10 |
| 25. Description of the coding tree | Did authors provide a description of the coding tree? | NA |
| 26. Derivation of themes | Were themes identified in advance or derived from the data? | 10 |
| 27. Software | What software, if applicable, was used to manage the data? | NA |
| 28. Participant checking | Did participants provide feedback on the findings? | 11 |
| Reporting |  |  |
| 29. Quotations presented | Were participant quotations presented to illustrate the themes / findings? Was each quotation identified? e.g. participant number | 16 to 26 |
| 30. Data and findings consistent | Was there consistency between the data presented and the findings? | 16 to 26 |
| 31. Clarity of major themes | Were major themes clearly presented in the findings? | 15 to 26 |
| 32. Clarity of minor themes | Is there a description of diverse cases or discussion of minor themes? | 15 to 26 |
|  |  |  |

Checklist extracted and adapted from:

Tong, A., Sainsbury, P., & Craig, J. (2007). Consolidated criteria for reporting qualitative research (COREQ): a 32-item checklist for interviews and focus groups. Int J Qual Health Care, 19(6), 349-357.
